# A Suppressor Mutation Partially Reverts the *xantha* Trait *via* Lowered Methylation in the Promoter of *Genomes Uncoupled 4* in Rice

**DOI:** 10.3389/fpls.2019.01003

**Published:** 2019-08-02

**Authors:** Meng Jiang, Yanhua Liu, Ruiqing Li, Yunchao Zheng, Haowei Fu, Yuanyuan Tan, Ian Max Møller, Longjiang Fan, Qingyao Shu, Jianzhong Huang

**Affiliations:** ^1^National Key Laboratory of Rice Biology, Institute of Crop Sciences, Zhejiang University, Hangzhou, China; ^2^Hubei Collaborative Innovation Center for Grain Industry, Yangtze University, Jingzhou, China; ^3^Institute of Nuclear Agricultural Sciences, Zhejiang University, Hangzhou, China; ^4^Jiaxing Academy of Agricultural Sciences, Jiaxing, China; ^5^Department of Molecular Biology and Genetics, Aarhus University, Aarhus, Denmark

**Keywords:** chlorophyll, DNA methylation, epigenetics, *genomes uncoupled 4*, photosynthesis, tetrapyrrole, rice (*Oryza sativa*)

## Abstract

The *xantha* trait of a yellow leaf rice mutant (HYB), controlled epigenetically by elevated CHG methylation of the *genomes uncoupled 4* (*OsGUN4*) promoter, has reduced chlorophyll content, altered tetrapyrrole biosynthesis, and deregulated transcription of photosynthesis-associated nuclear genes (PhANGs) compared to its wild-type progenitor Longtefu B (LTB). In the present study, we identified a suppressor mutant (CYB) of HYB and characterized its genetic, molecular, and physiological basis of the mutant phenotype. We found that the light-green phenotype of CYB was caused by a suppressor mutation in an unknown gene other than *OsGUN4*. Compared to HYB, the CHG methylation in the *OsGUN4* promoter was reduced, while OsGUN4 transcript and protein abundance levels were increased in CYB. The contents of total chlorophyll and its intermediate metabolites (except protoporphyrin IX) in CYB plants were intermediate between HYB and LTB. The expression levels of 30 genes involved in tetrapyrrole biosynthesis in CYB were all partially reverted to those of LTB, so were the PhANGs. In summary, a suppressor mutation caused the reversion of the *xantha* trait *via* reducing CHG methylation in *OsGUN4* promoter.

## Introduction

Chlorophyll, heme and other tetrapyrroles play key roles in photosynthesis (Mochizuki et al., 2010) and are therefore essential for all living organisms. 5-Aminolevulinic acid (ALA) is the first committed metabolite of tetrapyrrole biosynthesis and is synthesized from tRNA-bound glutamate by two reactions catalyzed in sequence by glutamyl-tRNA reductase (GluTR) and glutamate-1-semialdehyde aminotransferase (GSAT) in plants, algae, and most bacteria (Jahn et al., 1992; Tanaka and Tanaka, 2007). Many enzymes catalyze the chlorophyll biosynthesis from ALA to the final products, namely chlorophyll *a* and *b* in higher plants (Supplementary Figure S1; Beale, 2005; Tanaka and Tanaka, 2007). Mutations in any of these genes may reduce or even eliminate chlorophyll biosynthesis, leading to leaf color changes (Liu et al., 2007, 2016; Yoo et al., 2009; Tian et al., 2013; Wang et al., 2014; Zhang et al., 2015a; Yang et al., 2016).

*Genomes uncoupled 4* (*GUN4*) encodes an activator of Mg chelatase (MgCh) and interacts with the CHLH subunit (also known as GUN5) of MgCh and binds both the substrate protoporphyrin IX (Proto), and product Mg-Proto of the reaction catalyzed by MgCh (Larkin et al., 2003; Davison et al., 2005; Verdecia et al., 2005; Adhikari et al., 2009, 2011; Chen et al., 2015). GUN4 and GUN5 divert Proto from heme biosynthesis (Fe-Proto branch) to chlorophyll biosynthesis (Mg-Proto branch). Although not an enzymatic component of the tetrapyrrole biosynthesis pathway, GUN4 expression is indispensable under normal photoperiodic growth conditions (Larkin et al., 2003; Sobotka et al., 2008; Peter and Grimm, 2009; Brzezowski et al., 2014). GUN4 may be essential to activate ALA synthesis (Peter and Grimm, 2009). Phosphorylation of Ser located in the C-terminal extension of GUN4 was found to downregulate MgCh and provides an additional mechanism for the regulation of Mg-Proto branch biosynthesis (Richter et al., 2016). The *genomes uncoupled* (*gun*) mutants are impaired in the plastid-to-nucleus retrograde signaling (Larkin et al., 2003). A recent study indicated that ^1^O_2_ generated by GUN4-Proto could be a retrograde signal from the chloroplast to the nucleus (Tabrizi et al., 2016).

In a previous study, we generated a yellow-leaf (the *xantha* marker trait) mutant rice line Huangyu B (HYB) from the wild-type cultivar Longtefu B (LTB) by gamma irradiation (Zhou et al., 2006). Through gene mapping and additional studies, the mutant gene responsible for the *xantha* marker trait of HYB was identified as a mutated *OsGUN4* (*genomes uncoupled 4*) (Chi et al., 2010; Li et al., 2014). We further demonstrated the unique *xantha* marker trait of HYB is underpinned by an epiallele of the *OsGUN4,* which resulted from elevated CHG methylation in a 374-bp region (−2,497 to −2,124 bp) within the CpG island of the *OsGUN4* promoter. Remarkably, the ^−2386^C was completely methylated in HYB and was not methylated at all in LTB, and this ^−2386^C is part of an antioxidant response element (ARE) (Li et al., 2014). The epimutation almost abolished the transcription of *OsGUN4,* leading to significantly reduced chlorophyll biosynthesis (Li et al., 2014), altered tetrapyrrole biosynthesis and deregulated transcription of photosynthesis-associated nuclear genes (PhANGs) (Li et al., 2017).

To further understand the molecular mechanism leading to the epigenetic control of the *xantha* trait, we irradiated HYB with gamma rays to generate new mutations that could suppress the *xantha* marker trait phenotype by looking for revertant plants that are visually greener than HYB. Such phenotypic screening for suppressor mutants is well known for the discoveries of components in gibberellin signaling pathway in plants (Wilson and Somerville, 1995; Thomas and Sun, 2004; Sun, 2011). Both extragenic (Peng et al., 1999) and intragenic (Yamamoto et al., 2010) suppressor mutants involved in the GA (Gibberellin)–GID1 (Gibberellin Insensitive Dwarf1)–DELLA (Asp-Glu-Leu-Leu-Ala) signaling module were found (Sun, 2011). In the present study, we report the identification of such a suppressor mutant line (CYB), the genetic basis of the phenotypical mutation, and the effect of suppressor mutation on CHG methylation in the promoter of *OsGUN4* and consequently its expression, as well as on the biosynthesis of tetrapyrrole and transcription of PhANGs.

## Materials and Methods

### Plant Materials and Growth Condition

**Figure 1:**
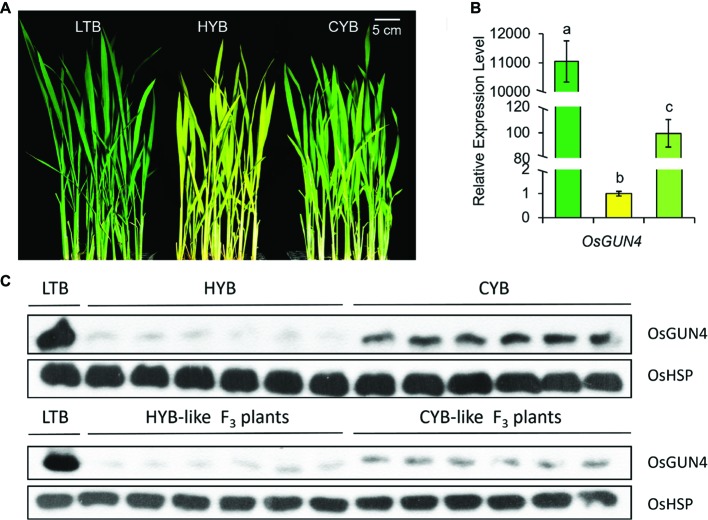
The phenotypes **(A)**, transcript abundances **(B)**, and protein levels **(C)** of OsGUN4 in LTB, HYB and CYB. **(B)** The expression levels were first normalized to the internal control gene *OsACTIN* and reported relative to each gene’s expression level of HYB (assigned a value of 1). Green, yellow, and light-green columns represent LTB, HYB, and CYB, respectively. All analyses were performed with six biological replicates. Error bars represent standard error. The different letters show the significant difference at a probability of *p* < 0.05. The data of LTB and HYB were already reported by Li et al. (2017). **(C)** Western blot assay of OsGUN4 expression was performed with heat shock protein (HSP) as an internal reference.

CYB was a mutant from HYB (*Oryza sativa* L. sp. *indica*) after irradiation of dry seeds with 200 Gy gamma rays, identified in a paddy field due to its light-green leaves that can be visually distinguished from the yellow ones of HYB plants (Figure 1A, Supplementary Figure S2; Fu, 2008). Genetic analysis was carried out in an F_2_ population developed from a cross of CYB and *O. sativa* sp. *japonica* cultivar Zaojing. For most in-door experiments, seedlings were first planted in barrels filled with paddy field soil and then transferred to a growth chamber at a photoperiod of 16 h of light (1,000 μmol photons m^−2^ s^−1^) at 30°C and 8 h of dark at 26°C.

### Determination of Chlorophyll and Tetrapyrrole Intermediates

Seedlings (32-day-old) were transferred to 1× MS liquid medium (Murashige and Skoog, 1962) for 3 days, and fresh leaf tissues were collected at midday for determination of chlorophyll, carotenoid, and tetrapyrrole intermediate contents according to Li et al. (2017). The chlorophyll, carotenoid and heme contents were determined according to the method described by Peter and Grimm (2009), and ALA content according to Czarnecki et al. (2012). For determination of Proto, Mg-Proto, and Protochlorophyllide *a* (Pchlide), approximately 500 mg fresh leaf samples were snap frozen in liquid nitrogen, homogenized, and mixed with 1 ml prechilled alkaline acetone (acetone: 0.1 N NH_4_OH; 9:1; v/v). The mixture was centrifuged at 16,000 × *g* for 5 min and the supernatant was used for determination of the three chlorophyll intermediates, using commercial enzyme-linked immunosorbent assay (ELISA) kit (Jingmei Tech., China). The assay is based on double-antibody sandwich-ELISA. The protocol for Proto assay is as follows. About 50 μl aliquots of supernatant with appropriate dilutions were added to microtiter plates precoated with Proto antibody and incubated at 37°C for 30 min. After thorough rinsing with washing solution, 50 μl of horseradish peroxidase (HRP)-labeled Proto antibody was added to the plates for a 30-min incubation at 37°C. After thorough rinsing, 3,3′,5,5′- tetramethylbenzidine (TMB) was added and incubated for 10 min at 37°C. The color reaction was terminated by 50 μl/well of acid termination solution, and the absorbance was measured within 15 min at 450 nm using a microtiter plate reader. Proto antibodies used in coating plate and in conjugation with HRP recognize different epitopes. Plates precoated with Proto antibody (as capture antibody), Proto standard, HRP-labeled Proto antibody, chromogen TMB, solutions for dilution, washing, and reaction termination were all provided with the kit. Content of Proto was calculated from standard curve using standard Proto provided with the kit following the same procedure as above. Similarly, Mg-Proto and Pchlide were determined using ELISA kits following the same protocol but with different pairs of antibodies.

### Quantitative Real-Time Polymerase Chain Reaction

Quantitative real-time polymerase chain reactions (qRT-PCRs) were performed for cDNAs suppressor transcribed from RNAs extracted from leaf tissues or protoplasts with the RNeasy Plant RNA Mini Kit (Qiagen, Hilden, Germany) using a SYBR Green GoTaq® qPCR Master Mix (Promega, WI, USA). The rice ACTIN gene (*Os03g0718100*) was used as an internal control, and relative expression levels were calculated using the 2^−ΔΔCt^ method (Livak and Schmittgen, 2001). Primers used for qRT-PCR are from Li et al. (2017).

### Bisulfite Sequencing

Bisulfite sequencing was used to identify methylated cytosines in *OsGUN4*. About 1 μg genomic DNA each was extracted from six 35-day-old rice seedlings. The DNA was treated with sodium bisulfite using the EZ DNA Methylation Kit (Zymo Research, Irvine, CA, USA) and the primers for amplification of *OsGUN4* (F: GGTTGGGAGTGTGTTTTAA, R: CAAAATAATACRCATAACAACAC) were designed by the Methyl Primer Express v1.0 (Applied BioSystems, USA) (Li et al., 2014). PCRs of the bisulfite-treated genomic DNAs were produced with EpiTaq HS polymerase (Takara, Dalian, China), and the PCR products were purified and cloned into the pGEM-T easy vector (Promega, Madison, WI, USA). At least 20 clones of each plant were sequenced and used for analysis of methylated cytosines with the Kismeth software (Gruntman et al., 2008).

### Western Blotting

For Western blotting, 200 mg of plant tissue of 35-day-old rice seedlings was ground in liquid nitrogen. The homogenate was resuspended for 60 min at 65°C in 1 ml extraction buffer [2% SDS, 12% sucrose, 50 mM dithiothreitol, 50 mM Na_2_CO_3_, 2 mM EDTA, pH 8.0, 0.1 mM phenylmethylsulfonylfluoride, and protease inhibitor cocktail (Sigma-Aldrich, St. Louis, MO, USA)]. The protein concentration was determined with the BCA Protein Assay kit (Thermo Scientific, Waltham, MA, USA). Antibodies against OsGUN4 (Li et al., 2014) was generated by BPI (Beijing Protein Innovation, Beijing, China) using the peptides FTRFFIRVGWMKKL. After separation by SDS-PAGE, the proteins were immunoblotted according to Li et al. (2014), using OsHSP (Os09g0482600) as a reference protein (Li et al., 2011).

### Statistical Analysis

Measurements of tetrapyrrole intermediates and qRT-PCRs were completed using six biological replicates, each consisting of pooled tissues from three seedlings. Measurements of bisulfite sequencing and Western blotting were completed using six individual plants. All statistical analyses were performed using the Student’s *t*-test and *F*-test.

## Results

### A Suppressor Mutation Partially Reverts the *xantha* Trait Toward Wild Type

We previously identified a light-green plant in an M_2_ population derived from gamma rays irradiated seeds of HYB in a paddy field. Subsequent cultivation demonstrated that the light-green revertant trait was stably inherited, and we named this mutant line as Cuiyu B (CYB). When grown in paddy fields, CYB, HYB, and wild type (WT) populations could be visually differentiated from each other, although it could be difficult to distinguish a few atypical individual seedlings (Figure 1A, Supplementary Figure S2).

To analyze the genetic nature of the revertant trait, we made reciprocal crosses between HYB and CYB and observed that the F_1_ plants all resembled CYB, with light-green leaves. In the HYB/CYB F_2_ population, there were two types of plants, i.e., light green and *xantha* with a segregation ratio of 3:1 ratio (917:266, *χ*^2^ = 3.86) (Table 1). We then produced a BC_1_ population of HYB/CYB/HYB and observed light-green and *xantha* plants segregated in a 1:1 ratio (43:34, *χ*^2^ = 1.05) (Table 1). All these results suggested that a dominant allele controls the light-green trait in the background of HYB.

**Table 1:** Segregation of plants with different leaf colors in various crosses between HYB, CYB, and wild-type line Zaojing.

Combination/generation	Green leaf	Light-green leaf	Yellow leaf	Ratio of segregation	*χ*2
CYB/HYB					
F_1_	0	12	0	–	
F_2_	0	917	266	3.45:1 (3:1)	3.86
HYB/CYB F_1_	0	33	0	–	
HYB/CYB//HYB BC_1_	0	43	34	1.26:1 (1:1)	1.05
Zaojing/CYB					
F_1_	11	0	0	–	
F_2_	1,015	183	72	14.1:2.54:1 (12:3:1)	17.6

We further crossed CYB with a wild type cultivar Zaojing, the F_1_ plants had leaves of normal green (as Zaojing). However, in its F_2_ population, we observed three types of plant: normal green (1015), light green (183), and yellow (as the *xantha* mutant) (72). The presence of three types of plant demonstrated that the light-green phenotype was caused by an additional mutation in an unknown gene other than *OsGUN4* because if it were due to a suppressor mutation of the *epi-gun4* that controls the *xantha* trait, then no *xantha* plants would have been observed in the F_2_ population of Zaojing/CYB (Table 1). Sequencing also demonstrated that there is no nucleotide difference in *OsGUN4* (including its promoter region) among CYB, HYB, and LTB (data not shown).

Based on the above observations, the light-green phenotype of CYB is likely to result from a suppressor mutation of the *xantha* mutation.

### Decreased Methylation in the *OsGUN4* Promoter Is Associated With the Reversion Phenotype

The epimutation of *OsGUN4* almost abolished its transcription in HYB (Li et al., 2014). We observed that transcription of *OsGUN4* was significantly increased in CYB than in HYB plants, though still far below that of LTB (Figure 1B). Western blot assay also confirmed that the level of translated OsGUN4 protein in CYB was higher than that in HYB but lower than that in LTB (Figure 1C).

**Figure 2:**
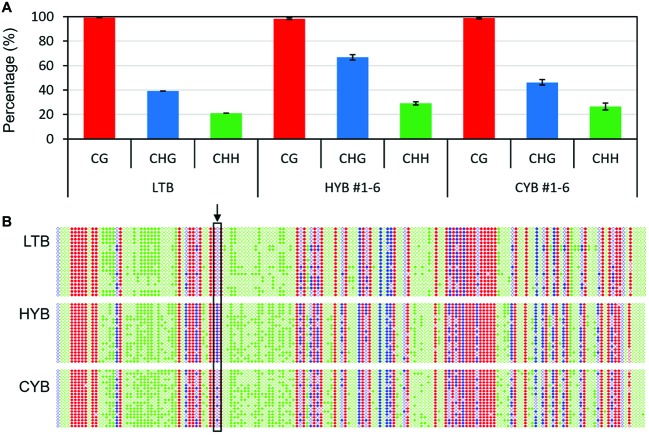
The cytosine methylation percentages **(A)** and profiles **(B)** in *OsGUN4* promoter in LTB, HYB, and CYB. **(A)** The cytosine methylation percentages in *OsGUN4* promoter region 2,497–2,124 bp upstream of the translation start site for CG (red), CHG (blue), and CHH (green) sites revealed by bisulfite sequencing. All analyses were performed with six biological replicates. **(B)** The cytosine methylation profiles of *OsGUN4* promoter region 2,497–2,124 bp upstream of the translation start site for CG (red), CHG (blue), and CHH (green) sites. H represents A, C, or T. The filled and empty circles denote methylated and unmethylated cytosines, respectively. The cytosine at position −2,386 is indicated by an arrow.

We then analyzed the methylation level and pattern in the 374-bp region within the CpG island (−2,497 to −2,124 bp), where the CHG methylation level was known to be significantly increased in HYB as compared to that of LTB (Li et al., 2014). Compared with HYB, the methylation level of CYB at CHG sites was decreased, while the methylation levels at CG and CHH sites were similar between HYB and CYB (Figure 2A). Remarkably, the ^−2386^C was completely methylated in HYB and was not methylated at all in LTB but was partly (20–30%) methylated in CYB (Figure 2B, Supplementary Figure S3).

To examine whether the epigenetic status was transmitted to offspring plants of HYB and CYB, we also analyzed six independent HYB- and CYB-like F_3_ plants derived from a cross of HYB and CYB, respectively. The methylation level for CYB at CHG sites was 43–50% compared with 64–79% for HYB (Figure 2A). Similarly, the methylation level of CYB-like F_3_ plants at CHG sites was 41–46% compared with that of HYB-like ones (63–72%) (Supplementary Figure S4).

These results not only support a link between decreased methylation of *OsGUN4* and the *xantha* suppressed phenotype but also demonstrate that the methylation change was meiotically heritable.

### The Suppressor Mutation Enhances Chlorophyll Biosynthesis With Partially Restored Transcriptional Regulation of Photosynthesis-Associated Nuclear Genes

**Figure 3:**
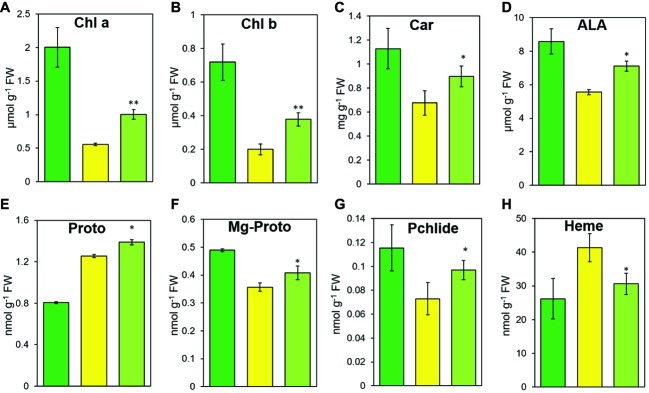
The steady-state levels of chlorophylls **(A,B)**, carotenoid **(C)**, and tetrapyrrole intermediates **(D–H)** in LTB (green), HYB (yellow), and CYB (light green). All analyses were performed with six biological replicates. Error bars represent standard error. * and ** represent significance between HYB and CYB mutant at *p* < 0.05 and *p* < 0.01, respectively. Chl *a*, chlorophyll *a*; Chl *b*, Chlorophyll *b*; car, carotenoid; ALA, 5-aminolevulinic acid; proto, Protoporphyrin IX; Mg-Proto, Mg-protoporphyrin IX; Pchlide, protochlorophyllide a.

Consistent with the leaf color change, CYB also had significantly higher chlorophyll *a*, chlorophyll *b*, and Car contents than HYB but still less than LTB (Figures 3A–C). Measurement of five tetrapyrrole intermediates revealed that the contents of all but Proto in CYB reverted toward the normal level found in the common progenitor line LTB (Figures 3D–H). HYB had significantly greater content of Proto than LTB (Li et al., 2017), CYB had an even greater level than HYB (Figure 3E).

**Figure 4:**
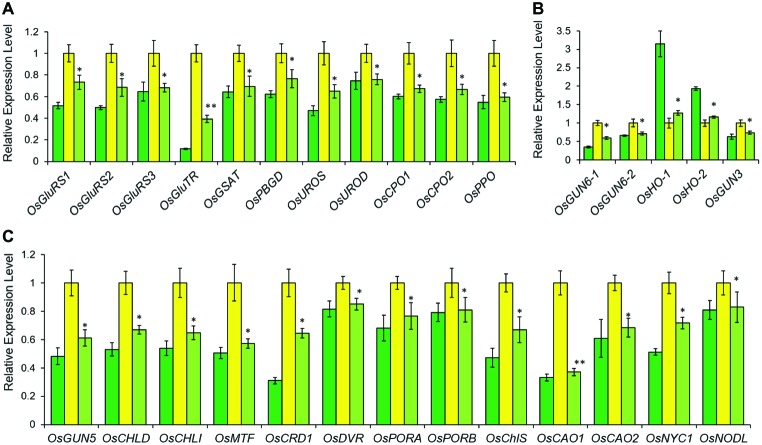
Relative expression levels of tetrapyrrole biosynthesis-associated genes in LTB (green), HYB (yellow) and CYB (light green). All analyses were performed with six biological replicates. The expression levels were first normalized to the internal control gene *OsACTIN* and reported relative to each gene’s expression level of HYB (assigned a value of 1). Error bars represent standard error. * and ** represent significance between HYB and CYB mutant at *p* < 0.05 and *p* < 0.01, respectively. **(A)** Transcript levels of the genes encoding enzymes for shared steps of the tetrapyrrole biosynthesis. **(B)** Transcript levels of the genes for the Fe-Proto branch. **(C)** Transcript levels of the genes for the Mg-Proto branch. All data of LTB and HYB were already reported by Li et al. (2017).

**Figure 5:**
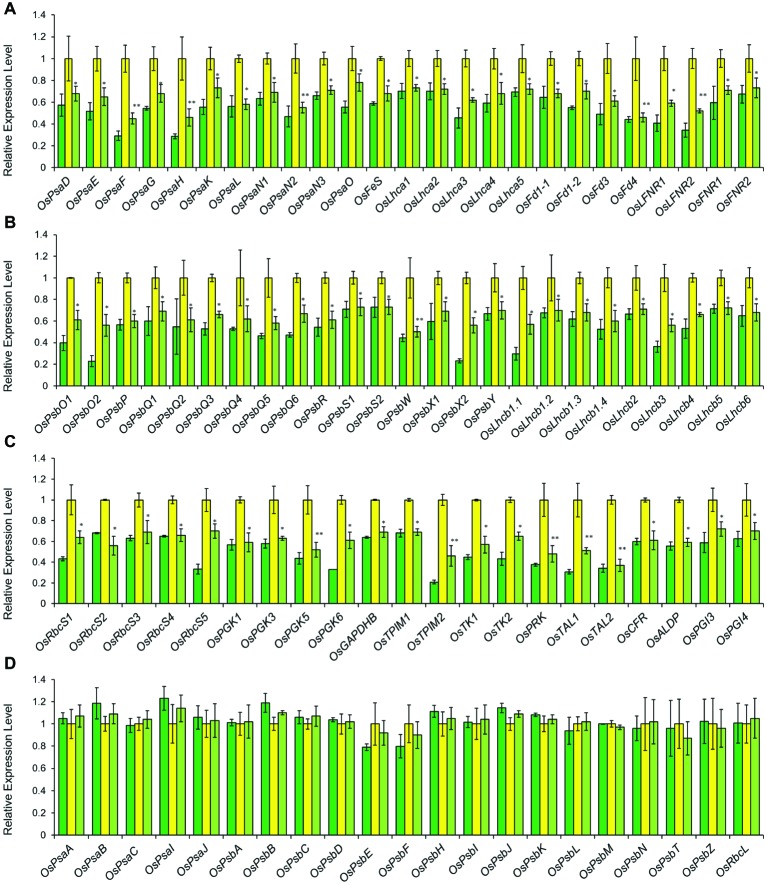
Relative expression levels of photosynthesis-associated nuclear genes (PhANGs) **(A–C)** and chloroplast genes (PhACGs) **(D)** in LTB (green), HYB (yellow) and CYB (light green). All analyses were performed with six biological replicates. The expression levels were first normalized to the internal control gene *OsACTIN* and reported relative to each gene’s expression level of HYB (assigned a value of 1). Error bars represent standard error. * and ** represent significance between HYB and CYB mutant at *p* < 0.05 and *p* < 0.01, respectively. **(A)** Transcript levels of PhANGs for PSI system; **(B)** Transcript levels of PhANGs for PSII system; **(C)** Transcript levels of PhANGs for CO_2_ fixation system; **(D)** Transcript levels of the PhACGs for PSI, PSII, and the CO_2_ fixation. All data of LTB and HYB were already reported by Li et al. (2017).

The abundance of *OsGUN4* transcripts was significantly increased in CYB plants as compared to that of HYB but was still far below that of LTB (Figure 1B). Consistent with its suppressed trait, CYB had levels of transcripts of 29 other genes, coding for enzymes in the tetrapyrrole biosynthesis pathway, all reverted from HYB toward those of LTB (Figure 4). Similarly, the expression levels of PhANGs in CYB were also reverted toward LTB (Figures 5A–C). On the other hand, there were no significant differences among LTB, HYB, and CYB regarding the transcript abundance of the photosynthesis-associated chloroplast genes (PhACGs) (Figure 5D).

## Discussion

We have here identified a suppressor mutation leading to a partial suppression of the *xantha* trait in rice (Figure 1A, Supplementary Figure S2). The suppressor mutation greatly decreased methylation of the *OsGUN4* promoter (Figure 2, Supplementary Figures S3, S4), which in turn leads to a greatly increased OsGUN4 expression (Figures 1B,C) and an enhanced rate of chlorophyll biosynthesis (Figures 3–5).

### The *xantha* Suppressor Mutation Is a Unique Genetic Resource for Understanding Epigenetic Variations

It has been speculated that epigenetic variation contributes to the overall genetic diversity in plants, but the extent to which epigenetic variation contributes to phenotypic variation in plants remains unknown because the number of epigenetically controlled, meiotically inheritable mutant alleles (epialleles) have been limited in plants (Weigel and Colot, 2013). Several cases where inherited phenotypic diversity resulted from epigenetic variation have been reported in rice: *epi-d1* (Miura et al., 2009), *epi-afo* (Wang et al., 2010), *epi-rav6* (Zhang et al., 2015b), and *epi-ak1* (Wei et al., 2017). Epigenetic variations can be generated naturally or by induction including use of mutagens; while several possible mechanisms leading to alteration of epigenetic status have been proposed, the actual mechanism leading to the generation of any epiallele remains elusive (Springer and Schmitz, 2017).

Further studies using the *xantha* suppressor mutant would provide insights into how the epigenetic variation is regulated and its potential contribution to phenotypic variation in plants. This would however await the cloning of the suppressor gene and disclosure of the underlying molecular mechanism leading to the decrease of CHG methylation in the *OsGUN4* promoter.

### The *xantha* Suppressor Mutation Stimulates Tetrapyrrole Biosynthesis

In the Mg-Proto branch, the Mg-Proto and Pchlide contents were significantly higher in CYB than in HYB (Figures 3F,G), similar to the increased abundances of all chlorophyll intermediates in transgenic tobacco plants overexpressing the *AtGUN4* gene (Peter and Grimm, 2009). OsGUN4 and AtGUN4 have similar effects on chlorophyll biosynthesis (Li et al., 2017). OsGUN4 promotes magnesium chelatase activity *in vitro* in rice (Zhou et al., 2012), but OsGUN4 will lose the function when its C terminal domain is absent (Zhou et al., 2012) or phosphorylated (Richter et al., 2016). Simultaneously, enhanced diversion of Proto to the chlorophyll biosynthesis decreases heme biosynthesis, which partially removes repression on GluTR and leads to increased ALA production. It meant that different *gun4* mutations could have different effects on chlorophyll biosynthesis. The increased abundance of Mg-Proto and Pchlide may be a prerequisite for the increased chlorophyll abundance in CYB (Figures 3F,G).

CYB accumulated significantly more ALA and Proto and significantly less heme than HYB plants (Figures 3D,E). The significantly increased transcript and protein levels of OsGUN4 in CYB would stimulate Mg-chelatase activity and consequently lead to an increase for Proto diverted to the Mg-Proto branch and a decrease in heme accumulation. The decreased heme level could explain the enhanced accumulation of ALA in HYB because heme and its catabolites repress ALA synthesis (Apitz et al., 2016).

In the tetrapyrrole biosynthesis pathway, there are 11 genes encoding proteins/enzymes for shared steps, 14 genes specific for the Mg-Proto branch and five genes specific for the Fe-Proto branch (Tanaka and Tanaka, 2007). The transcription of all tetrapyrrole biosynthesis genes except for *OsHO-1* and *OsHO-2* was significantly downregulated in CYB compared with HYB (Figure 4). The phenotypic suppression and partial restoration of chlorophyll biosynthesis in CYB are supported by the ~100-fold higher *OsGUN4* expression level as compared to HYB (Figure 1B). Although both transcription and translation levels of *OsGUN4* were significantly increased in CYB compared to HYB, their levels were still far below those of LTB and chlorophyll levels in CYB were only half of those in LTB (Figures 1B,C, 3A,B). This suggests that the OsGUN4 level in CYB is insufficient to support the full capacity of chlorophyll biosynthesis.

The transcriptional expression levels of PhANGs in CYB (Figures 5A–C) are closely associated with chlorophyll biosynthesis pathway through plastid-to-nucleus retrograde signaling mediated by *gun* genes (Chan et al., 2016). Similar to the tetrapyrrole biosynthesis genes, the transcripts of PhANGs was significantly lower in CYB than HYB, implying that the deregulation level of PhANGs was reduced in CYB (Figures 5A–C). This is consistent with reports for a *C. reinhardtii gun4* mutant (Brzezowski et al., 2014). At the same time, the transcript abundance of all the PhACGs was completely unaffected in HYB and CYB compared to LTB (Figure 5D), further supporting that PhANGs and PhACGs are regulated independently.

In summary, an additional mutation caused the reversion of the *xantha* trait in rice, through the suppression of elevated CHG methylation of the *OsGUN4* promoter. These findings are of importance for the understanding of diversity of epigenetic regulation and thus for the epigenetic manipulation of agronomic traits not only in rice but also in other crop plants.

## Data Availability

All datasets generated for this study are included in the manuscript and/or the Supplementary Files.

## Author Contributions

QS, MJ, and JH planned and designed the research. MJ, RL, YZ, YL, and YT performed the laboratory experiments, and MJ and HF did the field experiments. MJ, RL, JH and QS analyzed the data together. MJ finished the first draft, which JH, IM, LF, and QS edited and converted into the final draft. All authors reviewed and approved the final manuscript.

### Conflict of Interest Statement

The authors declare that the research was conducted in the absence of any commercial or financial relationships that could be construed as a potential conflict of interest.

## Supplementary Material

SUPPLEMENTARY FIGURE S1 Tetraphyrrole biosynthesis pathway in rice with detailed steps. The white typefaces represent the enzymes of tetrapyrrole biosynthesis pathway. The steps up to Proto, from which reactions are committed to either heme (Fe-Proto branch) or chlorophyll (Mg-Proto branch) biosynthesis, are shown in blue. The Mg-Proto branch and Fe-Proto branch are colored in green and orange, respectively. The rice genes of tetrapyrrole biosynthetic enzymes are shown in purple bold, while genes encoding HCAR and PBGS have not been found in rice are indicated in gray. Seven intermediates of tetrapyrrole biosynthesis measured in this study are shown in red bold. GluRS, glutamyl-tRNA synthetase; GluTR, glutamyl-tRNA reductase; GSAT, glutamate-1-semialdehyde aminotransferase; ALA, 5-aminolevulinic acid; PBGS, porphobilinogen synthase; PBGD, porphobilinogen deaminase; UROS, uroporphyrinogen-III synthase; UROD, uroporphyrinogen-III decarboxylase; CPO, coproporphyrinogen III oxidase; PPO, protoporphyrinogen IX oxidase; Proto, protoporphyrin IX; MgCh: protoporhyrin IX Mg-chelatase; ChlH, Mg-chelatase H subunit; ChlI, Mg-chelatase I subunit; ChlD, Mg-chelatase D subunit; Mg-proto, Mg-protoporphyrin IX; MTF, Mg protoporphyrin IX methytransferase; MTC, monomethyl ester oxidative cyclase; DVR, divinyl chlorophyllide a 8-vinyl reductase; POR: NADPH, protochlorophyllide oxidoreductase; ChlS, chlorophyll synthase; CAO, chlorophyllide a oxygenase; CBR, chlorophyll b reductase; HCAR, hydroxymethyl chlorophyll a reductase; FeCh, protoporhyrin IX Fe-chelatase; HO, heme oxygenase; PhS, phytochromobilin synthase; Pchlide, divinyl protochlorophyllide a.

SUPPLEMENTARY FIGURE S2 The phenotypes of LTB (green), CYB (light green), and HYB (yellow) in the field.

SUPPLEMENTARY FIGURE S3 Comparison of the cytosine methylation profiles in *OsGUN4* promoter region 2,497–2,124 bp upstream of the translation start site for CG (red), CHG (blue), and CHH (green) sites. The filled and empty circles denote methylated and unmethylated cytosines, respectively. The cytosine at position −2,386 is shown with an arrow.

SUPPLEMENTARY FIGURE S4 The cytosine methylation percentages in OsGUN4 promoter in HYB-like F_3_ plants and CYB-like F_3_ plants. The cytosine methylation percentages in OsGUN4 promoter region 2,497–2,124 bp upstream of the translation start site for CG (red), CHG (blue), and CHH (green) sites revealed by bisulfite sequencing.
