# Supplementary figures and images for: A Suppressor Mutation Partially Reverts the xantha Trait via Lowered Methylation in the Promoter of Genomes Uncoupled 4 in Rice
Source: Front Plant Sci. 2019 Aug 2;10:1003. doi: 10.3389/fpls.2019.01003 (PMC6688194; doi:10.3389/fpls.2019.01003)

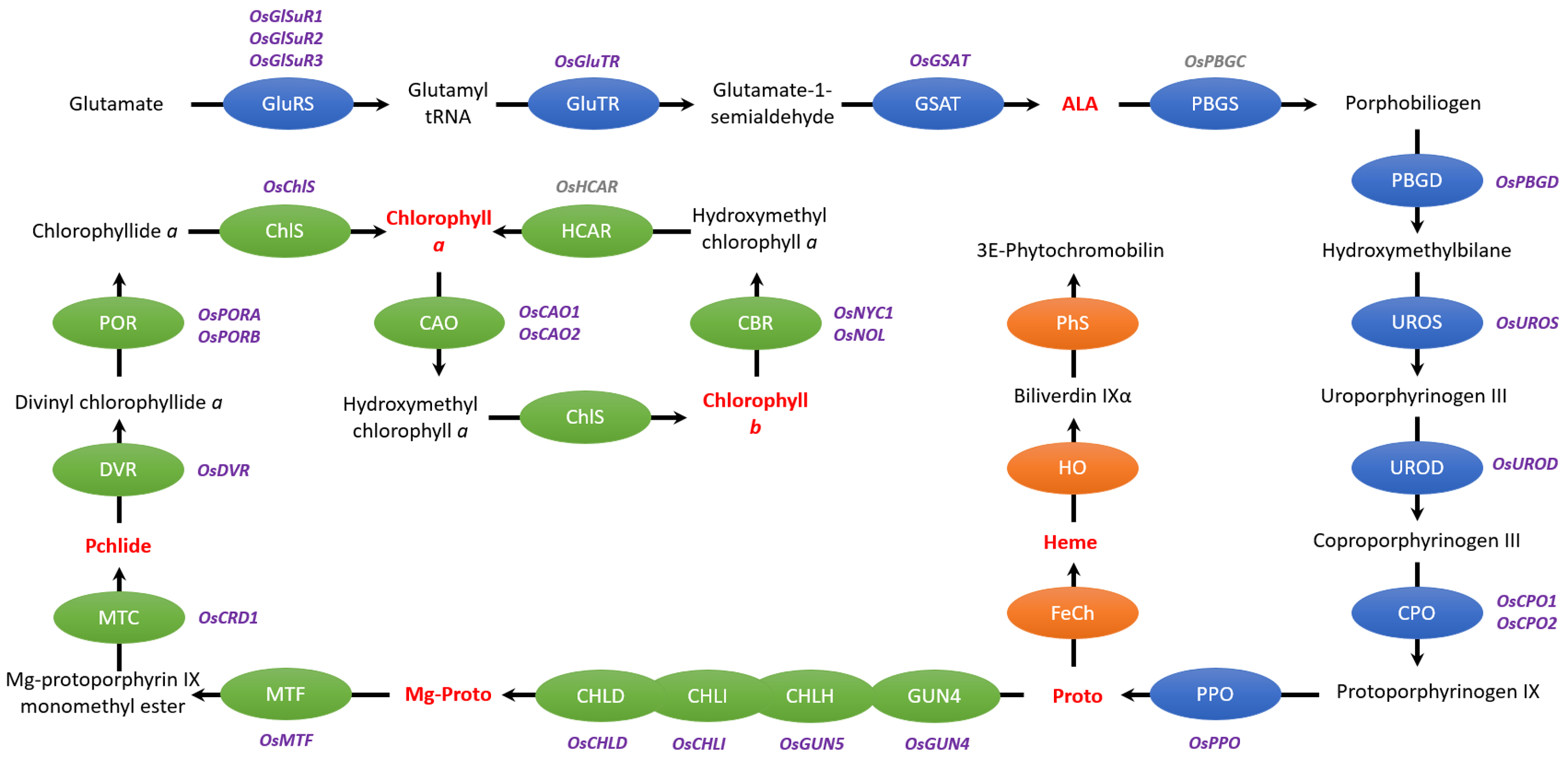

Supplement: SUPPLEMENTARY FIGURE S1 — Tetraphyrrole biosynthesis pathway in rice with detailed steps. The white typefaces represent the enzymes of tetrapyrrole biosynthesis pathway. The steps up to Proto, from which reactions are committed to either heme (Fe-Proto branch) or chlorophyll (Mg-Proto branch) biosynthesis, are shown in blue. The Mg-Proto branch and Fe-Proto branch are colored in green and orange, respectively. The rice genes of tetrapyrrole biosynthetic enzymes are shown in purple bold, while genes encoding HCAR and PBGS have not been found in rice are indicated in gray. Seven intermediates of tetrapyrrole biosynthesis measured in this study are shown in red bold. GluRS, glutamyl-tRNA synthetase; GluTR, glutamyl-tRNA reductase; GSAT, glutamate-1-semialdehyde aminotransferase; ALA, 5-aminolevulinic acid; PBGS, porphobilinogen synthase; PBGD, porphobilinogen deaminase; UROS, uroporphyrinogen-III synthase; UROD, uroporphyrinogen-III decarboxylase; CPO, coproporphyrinogen III oxidase; PPO, protoporphyrinogen IX oxidase; Proto, protoporphyrin IX; MgCh: protoporhyrin IX Mg-chelatase; ChlH, Mg-chelatase H subunit; ChlI, Mg-chelatase I subunit; ChlD, Mg-chelatase D subunit; Mg-proto, Mg-protoporphyrin IX; MTF, Mg protoporphyrin IX methytransferase; MTC, monomethyl ester oxidative cyclase; DVR, divinyl chlorophyllide a 8-vinyl reductase; POR: NADPH, protochlorophyllide oxidoreductase; ChlS, chlorophyll synthase; CAO, chlorophyllide a oxygenase; CBR, chlorophyll b reductase; HCAR, hydroxymethyl chlorophyll a reductase; FeCh, protoporhyrin IX Fe-chelatase; HO, heme oxygenase; PhS, phytochromobilin synthase; Pchlide, divinyl protochlorophyllide a. [file Image_1.TIFF]

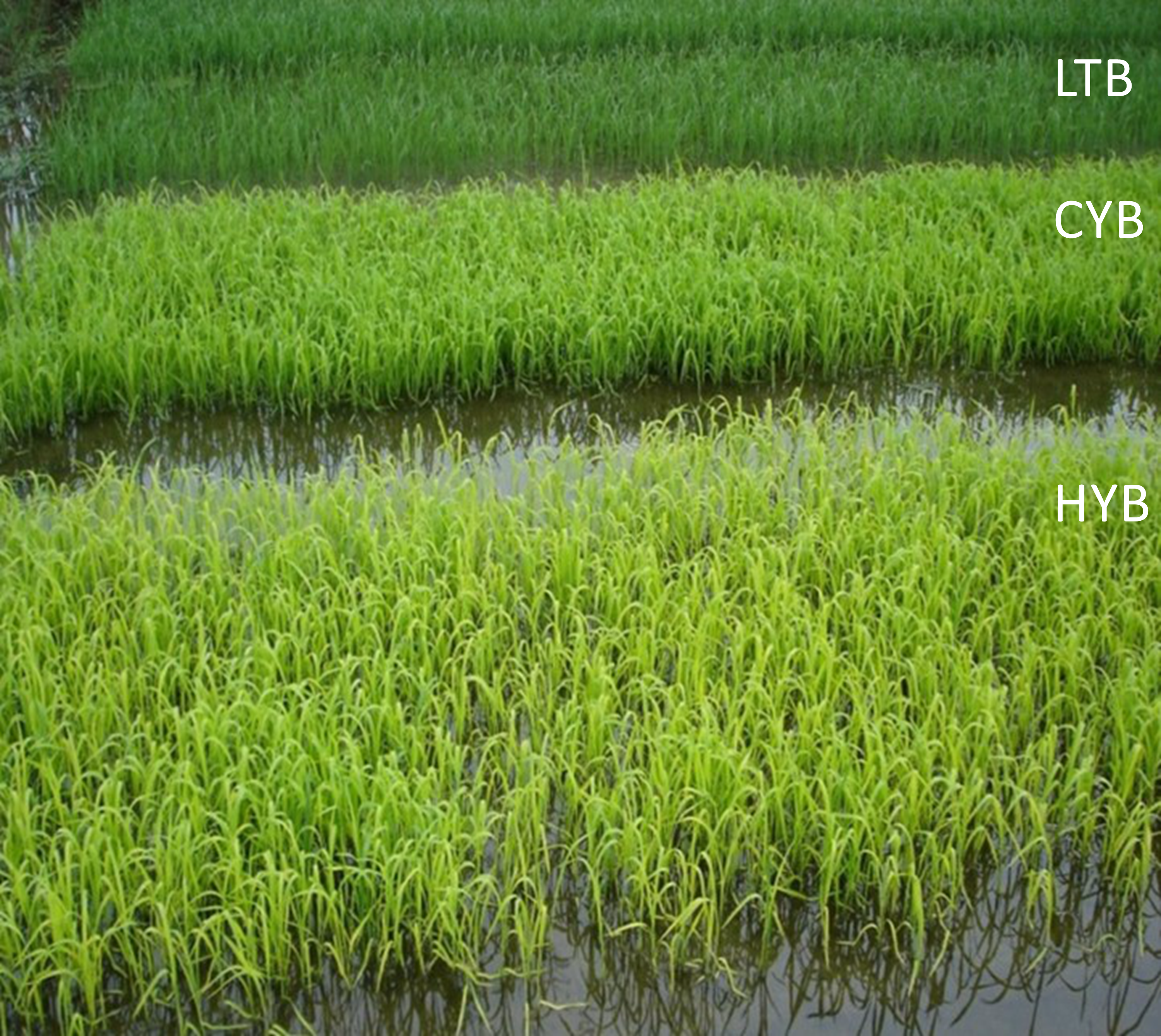

Supplement: SUPPLEMENTARY FIGURE S2 — The phenotypes of LTB (green), CYB (light green), and HYB (yellow) in the field. [file Image_2.TIFF]

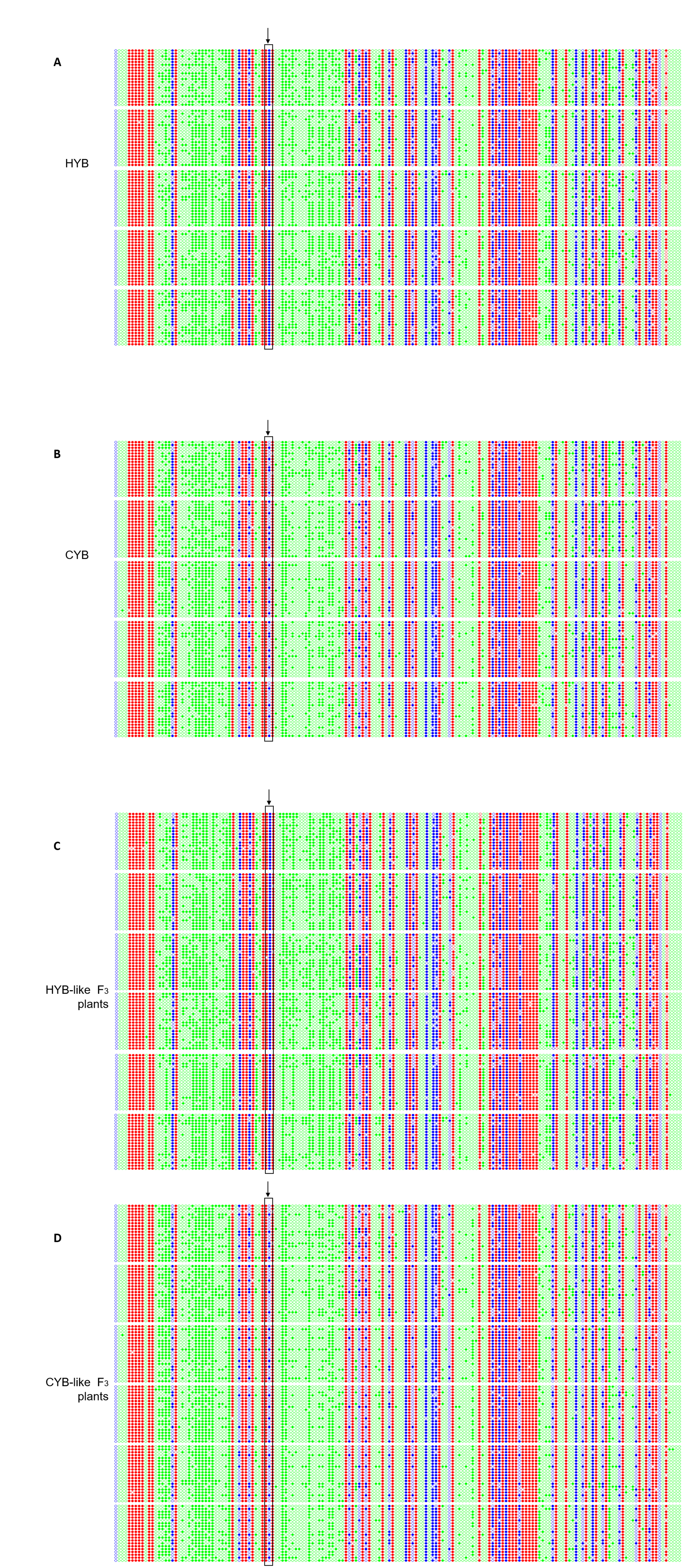

Supplement: SUPPLEMENTARY FIGURE S3 — Comparison of the cytosine methylation profiles in OsGUN4 promoter region 2,497–2,124 bp upstream of the translation start site for CG (red), CHG (blue), and CHH (green) sites. The filled and empty circles denote methylated and unmethylated cytosines, respectively. The cytosine at position −2,386 is shown with an arrow. [file Image_3.TIF]

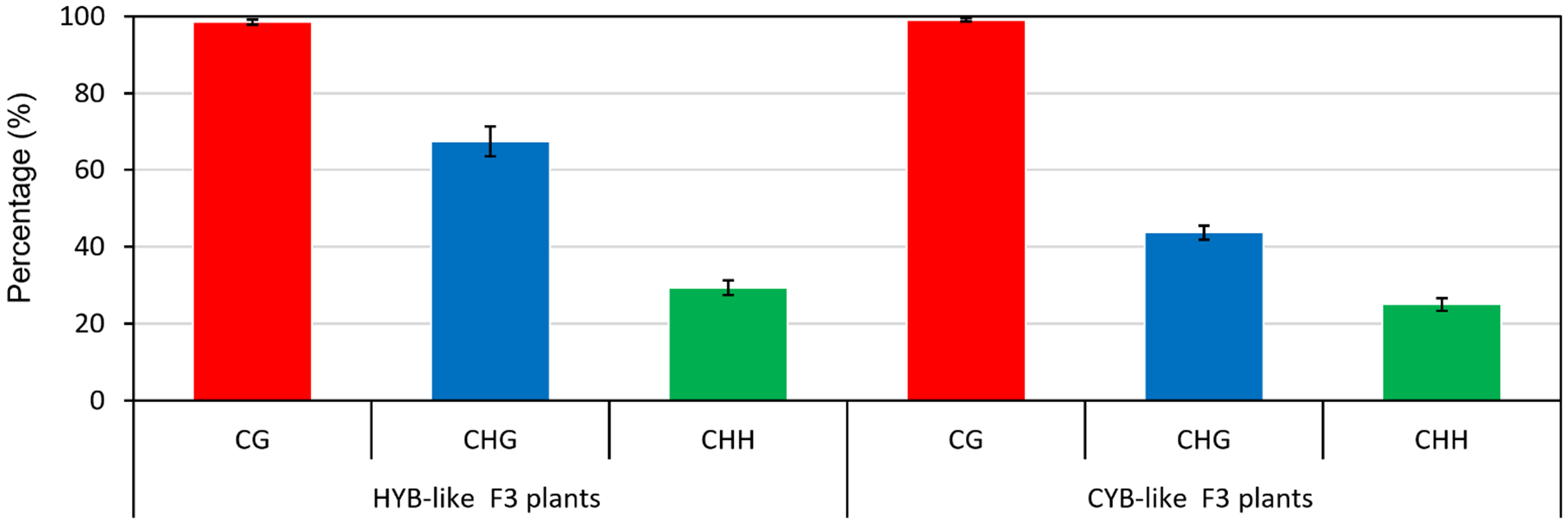

Supplement: SUPPLEMENTARY FIGURE S4 — The cytosine methylation percentages in OsGUN4 promoter in HYB-like F3 plants and CYB-like F3 plants. The cytosine methylation percentages in OsGUN4 promoter region 2,497–2,124 bp upstream of the translation start site for CG (red), CHG (blue), and CHH (green) sites revealed by bisulfite sequencing. [file Image_4.TIFF]
